# Supplementary material for: Resolving the full spectrum of human genome variation using Linked-Reads
Source: Genome Res. 2019 Apr;29(4):635–45. doi: 10.1101/gr.234443.118 (PMC6442396; doi:10.1101/gr.234443.118)
Supplement: Supplemental Material [file supp_gr.234443.118_Supplemental_longranger-2.2.zip › longranger-2.2.tar.bz2!/lr2.2-public/lib/stan/cmdstan/stan/doc/api/html/agrad_2rev_2functions_2log__diff__exp_8hpp_source.html]

Stan: src/stan/agrad/rev/functions/log\_diff\_exp.hpp Source File


|  |  |
| --- | --- |
|  | Stan  2.5.0  probability, sampling & optimization |


- Main Page
- Related Pages
- Namespaces
- Classes
- Files

- File List
- File Members

All Classes Namespaces Files Functions Variables Typedefs Enumerations Enumerator Friends Macros Pages

- src
- stan
- agrad
- rev
- functions

log\_diff\_exp.hpp

Go to the documentation of this file.

1 #ifndef STAN\_\_AGRAD\_\_REV\_\_FUNCTIONS\_\_LOG\_DIFF\_EXP\_HPP

2 #define STAN\_\_AGRAD\_\_REV\_\_FUNCTIONS\_\_LOG\_DIFF\_EXP\_HPP

3

4 #include <stan/agrad/rev/var.hpp>

5 #include <stan/agrad/rev/calculate\_chain.hpp>

6 #include <stan/agrad/rev/internal/vv\_vari.hpp>

7 #include <stan/agrad/rev/internal/vd\_vari.hpp>

8 #include <stan/agrad/rev/internal/dv\_vari.hpp>

9 #include <stan/agrad/rev/internal/vector\_vari.hpp>

10 #include <stan/agrad/rev/operators/operator\_greater\_than.hpp>

11 #include <stan/agrad/rev/operators/operator\_not\_equal.hpp>

12 #include <stan/math/functions/log\_diff\_exp.hpp>

13 #include <boost/math/special\_functions/expm1.hpp>

14

15 namespace stan {

16  namespace agrad {

17

18  namespace {

19  class log\_diff\_exp\_vv\_vari : public op\_vv\_vari {

20  public:

21  log\_diff\_exp\_vv\_vari(vari\* avi, vari\* bvi) :

22  op\_vv\_vari(stan::math::log\_diff\_exp(avi->val\_, bvi->val\_),

23  avi, bvi) {

24  }

25  void chain() {

26  avi\_->adj\_ += adj\_ \* calculate\_chain(avi\_->val\_, val\_);

27  bvi\_->adj\_ -= adj\_ / boost::math::expm1(avi\_->val\_ - bvi\_->val\_);

28  }

29  };

30  class log\_diff\_exp\_vd\_vari : public op\_vd\_vari {

31  public:

32  log\_diff\_exp\_vd\_vari(vari\* avi, double b) :

33  op\_vd\_vari(stan::math::log\_diff\_exp(avi->val\_, b),

34  avi, b) {

35  }

36  void chain() {

37  avi\_->adj\_ += adj\_ \* calculate\_chain(avi\_->val\_, val\_);

38  }

39  };

40  class log\_diff\_exp\_dv\_vari : public op\_dv\_vari {

41  public:

42  log\_diff\_exp\_dv\_vari(double a, vari\* bvi) :

43  op\_dv\_vari(stan::math::log\_diff\_exp(a, bvi->val\_),

44  a, bvi) {

45  }

46  void chain() {

47  bvi\_->adj\_ -= adj\_ / boost::math::expm1(ad\_ - bvi\_->val\_);

48  }

49  };

50  }

51

55  inline var log\_diff\_exp(const stan::agrad::var& a,

56  const stan::agrad::var& b) {

57  return var(new log\_diff\_exp\_vv\_vari(a.vi\_, b.vi\_));

58  }

62  inline var log\_diff\_exp(const stan::agrad::var& a,

63  const double& b) {

64  return var(new log\_diff\_exp\_vd\_vari(a.vi\_, b));

65  }

69  inline var log\_diff\_exp(const double& a,

70  const stan::agrad::var& b) {

71  return var(new log\_diff\_exp\_dv\_vari(a, b.vi\_));

72  }

73

74  }

75 }

76 #endif

dv\_vari.hpp

stan::agrad::log\_diff\_exp

fvar< T > log\_diff\_exp(const fvar< T > &x1, const fvar< T > &x2)

**Definition:** log\_diff\_exp.hpp:14

operator\_greater\_than.hpp

vd\_vari.hpp

stan::agrad::var::vi\_

vari \* vi\_

Pointer to the implementation of this variable.

**Definition:** var.hpp:40

stan::agrad::expm1

fvar< T > expm1(const fvar< T > &x)

**Definition:** expm1.hpp:15

log\_diff\_exp.hpp

vector\_vari.hpp

operator\_not\_equal.hpp

var.hpp

stan::agrad::calculate\_chain

double calculate\_chain(const double &x, const double &val)

**Definition:** calculate\_chain.hpp:8

stan::agrad::var

Independent (input) and dependent (output) variables for gradients.

**Definition:** var.hpp:27

calculate\_chain.hpp

vv\_vari.hpp


---

[ Stan Home Page ]

*© 2011–2014,
Stan Development Team.*
